# Eyeblink Synchrony in Multimodal Human-Android Interaction

**DOI:** 10.1038/srep39718

**Published:** 2016-12-23

**Authors:** Kyohei Tatsukawa, Tamami Nakano, Hiroshi Ishiguro, Yuichiro Yoshikawa

**Affiliations:** 1 Department of Systems Innovation, Graduate School of Engineering Science, Osaka University, 1-3 Machikaneyama, Toyonaka, Osaka, 560-8531, Japan; 2 Graduate School of Frontier Biosciences, Osaka University, 1-3 Yamadaoka, Suita, Osaka 565-0871, Japan; 3 JST ERATO Ishiguro Symbiotic Human Robot Interaction Project, Osaka University, 1-3 Machikaneyama, Toyonaka, Osaka, 560-8531, Japan

## Abstract

As the result of recent progress in technology of communication robot, robots are becoming an important social partner for humans. Behavioral synchrony is understood as an important factor in establishing good human-robot relationships. In this study, we hypothesized that biasing a human’s attitude toward a robot changes the degree of synchrony between human and robot. We first examined whether eyeblinks were synchronized between a human and an android in face-to-face interaction and found that human listeners’ eyeblinks were entrained to android speakers’ eyeblinks. This eyeblink synchrony disappeared when the android speaker spoke while looking away from the human listeners but was enhanced when the human participants listened to the speaking android while touching the android’s hand. These results suggest that eyeblink synchrony reflects a qualitative state in human-robot interactions.

Over the past several years, communication robots have become the locus of a considerable amount of interest. Such communication robots are expected to be used in a wide range of fields, such as department store customer service^1^,^2^ and even for some clinical functions^3^,^4^. Although the design and function of these robots will depend on their purposes, all of these robots will be developed to interact with humans. Because it has been shown that building rapport contributes to smooth interactions in human communication^5^, we believe it is important to establish some design principles that help build human-robot rapport.

In the case of human-human interaction, interpersonal synchrony—the coordination of behavior between communication partners—is known to contribute to establishing rapport^6^,^7^,^8^. This notion also holds true for some forms of human-robot/computer interactions as well. Several studies have reported that humans evaluated robots/virtual agents highly when these agents showed synchrony with humans^9^,^10^,^11^,^12^. Studies have also been conducted involving synchrony from humans to robots^13^,^14^ that showed that humans will synchronize with robots under certain conditions. However, there was a possibility that androids—humanoid robots with a human-like appearance—may not be accepted as a target of synchronous behavior due to certain negative characteristics that androids display, such as the “uncanny valley”^15^. For these androids to be recognized as reliable social entities, it is worth investigating whether humans show synchrony toward androids.

In human-human interactions, a receptive attitude toward a conversational partner is an important factor in facilitating synchrony. Bernieri *et al*. reported that when a pair of participants participated in a cooperative task, the ratings for synchrony and rapport were positively correlated. On the other hand, when the participants performed competitive tasks, this correlation was not observed^16^. Another study by Nagaoka *et al*. noted that displaying a receptive attitude leads to synchronization in response to the latency of dialogues^17^. Considering these studies, it appears that the degree of synchrony may be altered by biasing the conversation partner’s attitude in such a manner that he/she becomes receptive. We adopted this idea in the human-robot interaction context and examined the way in which biasing the conversation partner’s attitude affected the degree of synchrony by utilizing a robot that is capable of controlling its non-verbal actions.

However, no method has yet been established to reliably and quantitatively evaluate synchrony between humans and robots. In this regard, Nakano *et al*. introduced a method that quantitatively evaluates synchrony during human-human interactions by focusing on eyeblink synchrony^18^. These authors reported that eyeblink synchrony between a speaker and listeners occurred at the break points in speech but not during vocalization. Using this study as a model, in Experiment 1, we first examined whether eyeblink synchrony can be replicated between a human listener and an android speaker. Next, to examine whether the non-verbal expression of an android can bias humans’ attitudes and alter their degree of synchrony, we examined the manner in which the android’s gaze (when looking away from the human listener) can alter the degree of synchrony (Experiment 2). Because gaze is known to be capable of expressing mental states, such as affection and interest^19^, we hypothesized that gaze can also be used as a means of biasing the conversation partner’s attitude. Furthermore, in Experiment 3, we introduced touch as another means of generating alterations in attitude. Previous studies have demonstrated the manner in which touch can be used to improve an impression toward the toucher^20^,^21^. Therefore, we expected that touch would lead a listener to have a positive attitude regarding the android. In our experiment, the participants listened to the android’s speech while placing their hand on top of the android’s hand.

## Results

### Experiment 1: Human-Android Eyeblink Synchrony

**Figure 1:**
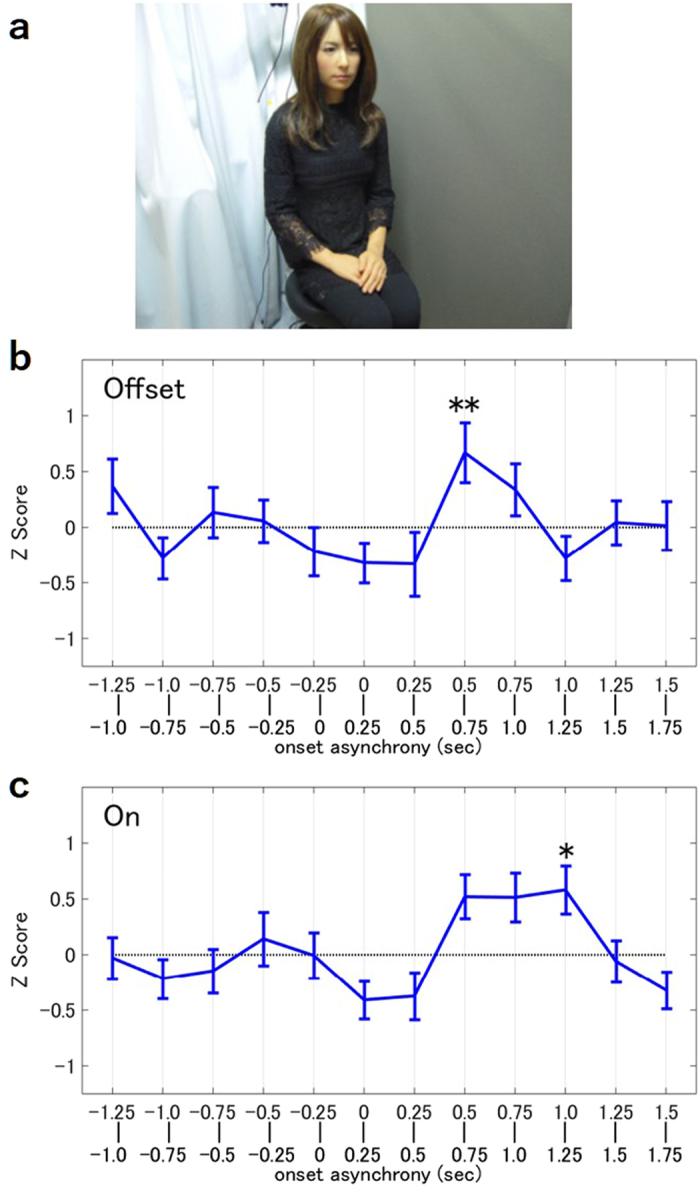
Experiment 1. (**a**) Geminoid-F: an android robot with a human-like appearance. (**b,c**) Eyeblink synchronization of the speaker in the Offset Condition and in the On Condition. The blue graph represents the mean of the blink frequency when transformed into Z scores. The difference over zero reflects the degree of eyeblink synchronization. The error bars illustrate the standard errors among the participants. (N = 27) *p < 0.05 **p < 0.01.

The first experiment was performed to examine whether eyeblink synchrony occurred between a human listener and an android speaker. Each participant listened to two speeches given by the android sitting in front of them (Fig. 1a). When the android blinked only at the breakpoints of speech (Offset Condition), the participants’ eyeblink rate significantly increased after the android’s eyeblinks with a delay of 0.5~0.75 sec (Fig. 1b, z = 3.47, p < 0.01, one sample Z test). By contrast, when the android blinked during vocalization (On Condition), a significant increase in the listeners’ eyeblink rate was observed 1.0~1.25 sec after the android’s eyeblink (Fig. 1c, z = 3.02, p < 0.05, one sample Z test).

### Experiment 2: Biasing Human Attitude with Gaze

**Figure 2:**
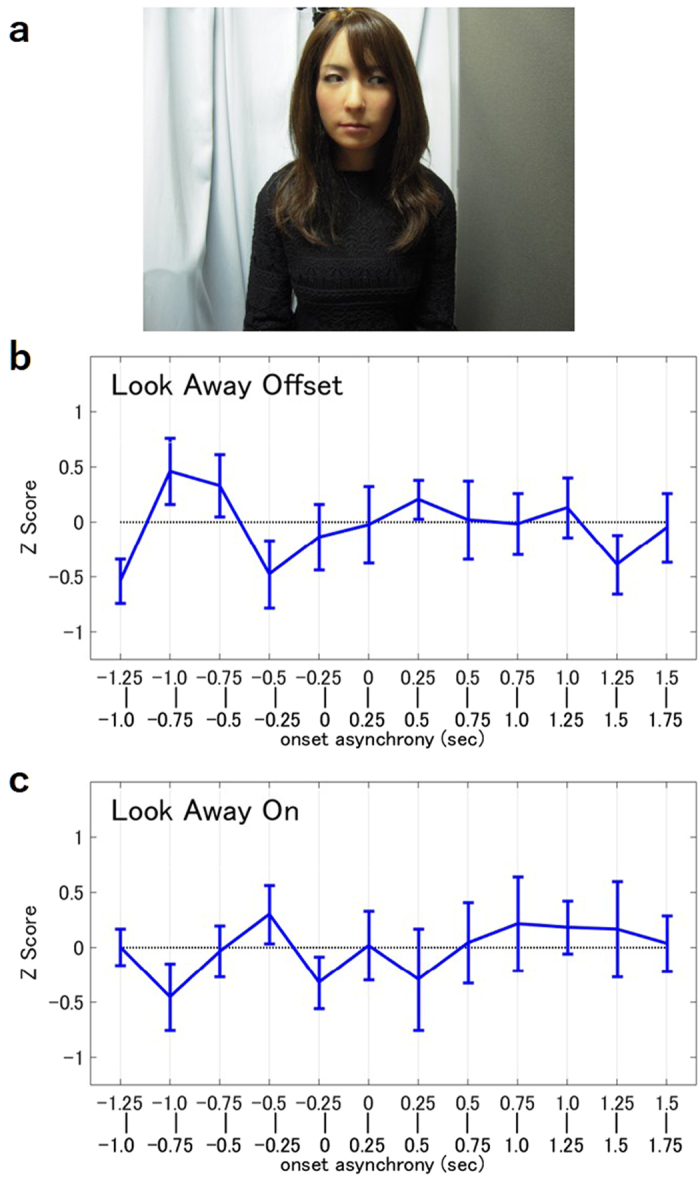
Experiment 2. (**a**) Android’s gaze during Experiment 2. (**b,c**) Eyeblink synchronization of the speaker in the Look Away Offset Condition (N = 14) and the Look Away On Condition (N = 12).

The second experiment was performed to examine whether shifting the android’s gaze away from the listener affects the degree of eyeblink synchrony. In this experiment, the android gave a speech while directing its gaze away from the human listener throughout (Fig. 2a). In contrast with Experiment 1, a significant increase in the listeners’ eyeblink rate was not observed at any time point, regardless of whether the android blinked at the breakpoints of the speech (Fig. 2b) or during vocalization (Fig. 2c).

### Experiment 3: Biasing Human Attitude with Touch

**Figure 3:**
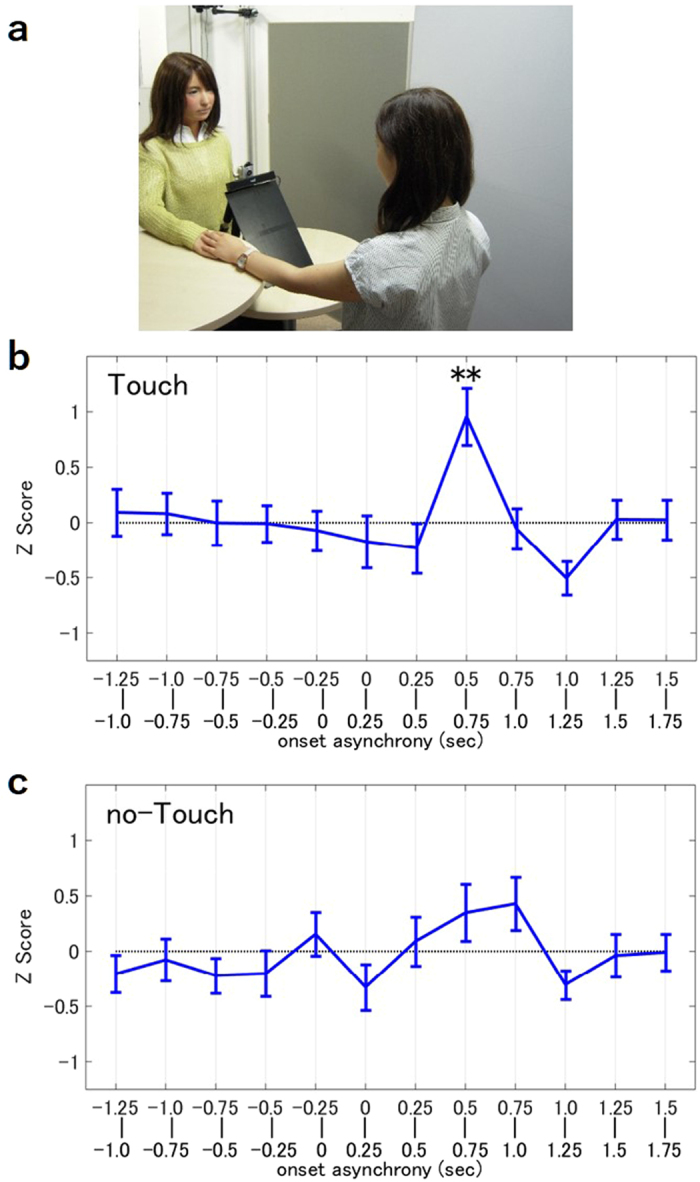
Experiment 3. (**a**) The participant touching the android’s hand. (**b,c**) Eyeblink synchronization of the speaker in the Touch Condition and in the No-Touch Condition. (N = 30) **p < 0.01.

In Experiment 3, we investigated whether touch has any influence on the degree of eyeblink synchrony. In this experiment, a human listener listened to two speeches, one while putting his hand on top of the android’s (Touch Condition), as shown in Fig. 3a, and another without touching the android (No-Touch Condition). In the Touch Condition, a significant increase in the listeners’ eyeblink rate was observed 0.5~0.75 seconds after the android’s eyeblink (Fig. 3b, z = 5.25, p < 0.01, one sample Z test). Conversely, there was no significant increase in the eyeblink rate in the No-Touch Condition (Fig. 3c). The listeners’ eyeblink rate 0.5~0.75 sec after the android’s eyeblink in the Touch Condition was higher than the rate in the No-Touch Condition but did not reach the level of significance (t = −1.88, p = 0.07, paired t test).

## Discussion

The present study clearly shows that eyeblink synchrony does occur between human listeners and android speakers in face-to-face interaction. Now that we know that the degree of eyeblink synchrony can be quantitatively evaluated, we will consider utilizing this metric to evaluate how firm rapport is established. To do so, we will need to further investigate the degree to which eyeblink synchrony contributes to building rapport. Recently, Nomura & Kanda have published a scale to evaluate human-robot rapport^22^, which may be useful in investigating the extent to which eyeblink synchrony contributes to building rapport.

We conducted Experiments 2 and 3 to investigate whether the nonverbal features that the android expresses generate a bias in humans’ attitudes, thus leading to an alteration in the degree of eyeblink synchrony. The results of these two experiments showed that our conjecture appears to be correct. When the android displayed an unpleasant expression—when looking away from the human listener—the synchronous eyeblinking in humans was inhibited. We presume that this is the case because the shift in the gaze induced a non-receptive attitude. In contrast, when the human listener touched the android’s hand, an action aimed at biasing the listener’s attitude toward receptivity, the degree of eyeblink synchrony was higher than that in the No-Touch Condition. These results are in line with those of a previous study that has shown how a receptive attitude toward others leads to synchronous behavior^17^. Furthermore, Bernier *et al*. have noted that synchrony and rapport are positively correlated when interactions are evaluated under a receptive attitude^16^. Therefore, there is a possibility that rapport may have been established more strongly under the Touch Condition. To further understand the relationships among receptive attitude, synchrony, and rapport, an additional experiment involving psychological evaluations may be of value.

Notably, although Nakano *et al*. have reported that eyeblink synchrony between listeners and speakers occurs at the breakpoints of the speech but not during vocalization, an increase in the listeners’ eyeblink rate was observed in the present study not only during the offset of vocalization but also during vocalization. We speculate that the increase in eyeblink rate during vocalization has more to do with mimicry than synchrony. Nakano *et al*. have suggested that the eyeblink synchrony observed in their study was more related to behavioral coordination at speech junctions rather than the chameleon effect, in which a mere perception of another’s behavior triggers an unconscious mimicry^7^. In the present study, the android was controlled to move its eyelids and mouth but not any other body part, whereas in the previous research, the video speaker made various movements. We presume that because the android’s eyeblink attracted more attention and because the listeners’ choice of which modality to mimic was limited in our study, it was effective in triggering the eyeblink mimicry. Another possible distinguishing feature may involve the difference in the type of media used to display the speaker’s eyeblinks (3D vs. 2D). A previous study has shown that trust is established more quickly when humans interact face-to-face (3D) rather than through video conferencing (2D)^23^. Because Lakin and Chartland have stated that mimicry may increase when a person has a desire to affiliate^24^, eyeblink mimicry may have been more effectively induced by the android speaker (3D) compared with Nakano *et al*.’s 2D video of a speaker. Further investigation is necessary to confirm this conclusion.

In Experiment 3, the setting of the No-Touch Condition was almost identical to the Offset Condition of Experiment 1. However, a contrasting result was observed in these conditions. Although a significant increase in the listeners’ eyeblink rates was observed in the Offset Condition, this increase was not observed in the No-Touch Condition. We suspected that the inconsistency of the results was caused by the difference in the distances between the listener and the android. According to a study on human proximity, 120 cm is the approximate border of personal space that should not be violated by non-friends^25^. From another previous study on personal space, it is known that the intrusion of personal space can cause discomfort in human-human interactions^26^ as well as in human-robot interactions^27^. The distances between the listener and android in our experiments were approximately 120 cm in Experiment 1 and 80 cm in Experiment 3. We presume that the distance was too small in the No-Touch Condition, such that it invaded the listener’s personal space and created discomfort. Under the assumption that a person’s attitude can affect the occurrence of synchrony into account^17^, the feeling of discomfort may have biased the listeners’ attitude toward being non-receptive and may have led to the inhibition of synchrony. To further investigate the effect of proximity on the degree of synchrony, it would be worthwhile to test our experiment using a variety of distances and to evaluate the stress of participants to confirm the links across distance, discomfort and synchrony.

To establish eyeblink synchrony as a widely applicable way to evaluate rapport, we would need to investigate whether the eyeblink synchrony seen in this work can be replicated using other robots that do not closely resemble humans. It still remains unclear what feature of the android was instrumental in obtaining the present results. It would also be valuable to conduct the same experiment task using a human speaker so that we could better compare how participants react to a human speaker versus an android speaker.

As mentioned in the introduction, synchrony has a connection with rapport^6^,^7^,^8^. To establish strong rapport, it would be valuable to further investigate ways to synchronize more often. Ford *et al*. have reported a co-occurrence between eyeblinking and other non-verbal communicative behaviors, such as gaze and facial expression^28^. Thus, we may be able to predict when humans will exhibit eyeblinks, thus providing the android more opportunities to synchronize its eyeblinks. It would be valuable to test this idea by supplying our android with many different motions and to observe how human’s eyeblinks would behave. Additionally, we suspect that by adding a number of other motions and modalities, the number of channels for synchrony to occur would expand, and the interplay of multiple motions/modalities might help to induce further synchrony. Therefore, we believe that this possibility should be examined by analyzing human-robot interactions in which the robot has implemented multiple motions and modalities, to investigate how synchrony and different motions/modalities relate to one another in detail.

In summary, we investigated how synchrony between humans and androids is influenced by biasing humans’ attitudes. In Experiment 1, we examined whether the eyeblink synchrony between the human listener and human speaker in a video shown in Nakano *et al*. can be replicated between a human and android in the presence of one another. The results revealed that the listener’s eyeblink rate increased directly after the android’s eyeblinks, which occurred at the breakpoints of speech. This result is in line with the results of Nakano *et al*., indicating that the listener is likely to perform eyeblinks at a consistent rate not only for human speakers but also for android speakers. Additionally, the increase of the listener’s eyeblink rate was also observed immediately after the android’s eyeblinks during vocalization, although this increase was not observed in the previous study, which implies that humans may have a tendency to unconsciously mimic the android’s eyeblink. We then investigated the effects of the non-verbal expression that the android has on the degree of synchrony. The results show that when the android looked away from the listener, eyeblink synchrony was inhibited, whereas when the listener made physical contact with the android, the listener was more likely to exhibit synchronous eyeblinks. These results suggest that some non-verbal expressions that an android display may serve as a powerful means of biasing human attitudes, leading to an alteration in the degree of synchrony. It is probable that there are many other ways to bias human attitudes. Therefore, we believe it is important to seek out other non-verbal expressions for robots that contribute to inducing synchrony.

## Method

### Participants

Thirty-three participants (14 males and 19 females) were recruited for Experiment 1. Four participants were excluded from the analysis due to their failure to detect eyeblinks. The mean eyeblink rate at this point was 32.1 min^−1^ (SD = 15.5). Two other participants were excluded from the analysis because of excessive eyeblink rates (two standard deviations away from the mean). Consequently, we analyzed the data acquired from 27 participants (12 males and 15 females). The participants’ mean age was 21.1 years old, and the mean eyeblink rate was 29.5 min^−1^.

Experiment 2 utilized the same 33 participants who were recruited for Experiment 1. Of the 33 participants, 6 participants were excluded from the analysis due to a failure to detect eyeblinks and 1 participant was excluded due to an excessively high eyeblink rate. Thus, 26 participants remained (12 males, 14 females, mean age of 21.0 years), and their mean eyeblink rate was 31.2 min^−1^.

For Experiment 3, 32 new participants were recruited. Of these, 2 participants were excluded from the analysis, one due to a failure in eyeblink detection and another for an excessive eyeblink rate. The mean eyeblink rate of the 30 participants (14 males, 16 females) was 32.8 min^−1^.

All participants gave written informed consent. The study was approved by the Graduate School of Engineering Science at Osaka University and the methods were carried out in accordance with the approved guidelines.

### Android

In this experiment, we used Geminoid-F (Fig. 1a), a female android resembling an actual person in appearance. Geminoid-F has a total of 12 pneumatic actuators embedded inside its body. Each actuator is activated by channeling air from an external air compressor. The motion of Geminoid-F is controlled by receiving signals indicating the desired positions of the actuators that are sent from an external computer at a sampling rate of 20 Hz. Various motions can be generated by changing the values of the signals. The generated motions can be saved in a text file and used to reproduce the same motions. We call this file the Motion File. In this experiment, we generated the motions using only the eyelid and mouth, while the remaining actuators were set as static. In addition, the motion for shifting the android’s gaze away from the participants was generated for the second experiment.

The mouth motion was generated using a conventional method for approximating the speaker’s mouth motion based on the voice^29^. To create the appearance of speech for Geminoid-F, the sound of the speech was played in sync with the generated mouth motion. The sound was broadcast by an external audio speaker that was set directly behind Geminoid-F. Three speeches were used in this experiment, all of which were acquired from a Japanese radio program in which a female radio personality speaks. The lengths of the speeches were: Speech A = 225 sec, Speech B = 227 sec, Speech C = 227 sec. The lengths were edited to match the lengths used in Nakano *et al*.’s study as closely as possible^18^. Speeches A and B were used in the first and third experiments. Speech C was used in the second experiment. We additionally edited a speech (85 sec) from the same radio program to be used in a practice session for the experiments.

We generated two eyeblink motions, one that occurred only at the breakpoints of speech and another that occurred only during vocalization. To generate eyeblink motions that occurred only at the breakpoints of the speech, we defined the timing of the breakpoint of speech as “50 msec before the start of interval of time when Geminoid-F’s mouth is closed for more than 400 msec.” By timing the eyeblinks according to the above definition, the total eyeblinks during each speech was 54 (Speech A), 66 (Speech B) and 56 sec (Speech C). To generate an eyeblink motion during vocalization, eyeblinks were set to occur when Geminoid-F’s mouth was in motion to avoid blinking at the breakpoints of speech. The number of eyeblinks in the On Condition was the same as in the Offset Condition.

### Data Acquisition

Vertical electrooculograms (EOGs) were used to measure participants’ eyeblinks. The electric potential difference between the cornea and retina that were caused by the eyeblinks were recorded using two active surface electrodes attached above and below the eye. POLYMATE2 of the TEAC Corporation was used to record the EOG at a sampling rate of 1000 Hz.

### Procedure

Experiment 1 was conducted one participant at a time. Each participant was instructed to listen to the three speeches given by the android. After listening to each speech, they were asked to answer a few simple questions regarding the content the speech. They were also informed that their eye movements would be recorded but were unaware that their eyeblinks were being recorded. The participants were asked to sit in front of Geminoid-F (Fig. 1a), a female android, and electrodes were attached to their faces to record their eyeblinks. When the machine was ready to record, the participants listened to the three speeches. The first speech was given as a practice round. The participants listened to a short speech given by the android and answered a few multiple choice questions. The android’s eyeblinks were regulated to occur once every three seconds at random. The second and third speeches and quizzes were performed using the same procedure. The timing of the android’s eyeblinks during the second and third speeches were timed to occur only at the breakpoints of the speech (Offset Condition) or while vocalizing (On Condition). The combination of the order and the two conditions were counterbalanced across participants such that each participant experienced both the Offset and On Conditions. The mean score of the quizzes was 98.7% (SD = 2.6%), suggesting that the participants were focused on the speeches and understood the content.

Experiment 2 was conducted with the same participants and performed in the same setting as the previous experiment, directly after they finished answering the third quiz. Participants listened to the speech given by the android in either the Look Away Offset Condition, in which the android blinked only at the breakpoints of the speech, or the Look Away On Condition, in which the android blinked only during vocalization. The android shifted its gaze away from the participant a few seconds before it began to speak and maintained this position throughout the speech. A multiple choice quiz was given after the speech, and the results showed that the participants understood the content of the speeches well (mean score = 98.0%, SD = 5.3%).

In Experiment 3, the setting and procedure were identical to Experiment 1, except for the manner in which the participants listened to the second and third speeches. The participants were instructed to listen to the speeches under two conditions, the Touch Condition and the No-Touch Condition. In the Touch Condition, the participants placed their hand on top of the android’s hand while listening to the speech (Fig. 3a). In the No-Touch Condition, the participants listened to the speech without touching the android’s hand, which served as a control condition. In both conditions, the android’s eyeblinks were fixed to occur only at the breakpoints of speech. The order of the two conditions were counterbalanced across participants. The mean score of the quizzes was 97.7% (SD = 3.9%), once again suggesting that the participants were focused on the speeches.

### Data Analysis

We followed the data analysis method used in Nakano *et al*.^18^.

The participants’ eyeblink onset times were detected by processing the EOG signals^30^. Geminoid-F’s blink onset times were detected by referring to the Motion File.

To investigate the degree of synchrony between the android’s and the participants’ eyeblinks, we examined the linkage of the participants’ eyeblink rate with the android’s eyeblinks (1.25 sec before and 1.75 sec after the eyeblink onset). We divided this interval into 12 equally sized bins and counted the number of participants’ eyeblinks that occurred in each bin. If there was a significant increase in the participants’ eyeblink rate in a specific bin, it can be interpreted that the android’s and the participants’ eyeblinks occurred in synchrony. To examine their relation, we conducted a one-sample Z test and compared the observed participants’ eyeblink rate in the experiments and the eyeblink rate that occurred at random.

We randomized the time series of the observed eyeblinks by shuffling the inter-blink intervals, generating 1000 pieces of surrogate data. These surrogate data can be considered the participants’ eyeblink rate that occurred by chance^31^. Using these surrogate data in the same procedure described above, we counted the number of generated eyeblinks that occurred in relation to the android’s eyeblinks and calculated the sum and standard deviation of the numbers in each bin. The number of observed eyeblinks was than transformed to a Z score using the mean and standard deviation of the numbers derived from the surrogate data. The same procedure was performed for the data acquired from all participants, thus generating a Z score from each participant. To examine whether the observed eyeblink rate was greater than the level of chance, we took the Z scores of each bin and applied a one-sample Z test. Bonferroni corrections were applied to generate multiple comparisons.

## Additional Information

**How to cite this article**: Tatsukawa, K. *et al*. Eyeblink Synchrony in Multimodal Human-Android Interaction. *Sci. Rep.* **6**, 39718; doi: 10.1038/srep39718 (2016).

**Publisher's note:** Springer Nature remains neutral with regard to jurisdictional claims in published maps and institutional affiliations.
